# Moringa oleifera extract (Lam) attenuates Aluminium phosphide-induced acute cardiac toxicity in rats

**DOI:** 10.1016/j.toxrep.2018.01.001

**Published:** 2018-01-28

**Authors:** Ahmed S. Gouda, Nagla A. El-Nabarawy, Samah F. Ibrahim

**Affiliations:** a National Egyptian Center of Environmental and Toxicological Research, Faculty of Medicine, Cairo University, Egypt; b Forensic and Toxicology Department Faculty of Medicine, Cairo University, Egypt; c Clinical Departement, Princess Nourah Bint Abdulrahman University, Riyadh, King Saudi Arabia

**Keywords:** AlP, aluminium phosphide, Lam, moringa oleifera extract, CAT, catalase, GR, glutathione reductase, SOD, superoxid dismutase, MDA, malondialdehyde (product of lipid peroxidation), ROS, reactive oxidative stress, Toxicity, Aluminium phosphide, Cardiac toxicity, Oxidative stress, Moringa oleifera extract

## Abstract

**Background:**

Moringa oleifera extract (Lam) has many antioxidant and protective properties. Objective: to investigate the antioxidant activities of Lam in counteracting the high oxidative stress caused by acute sub-lethal aluminium phosphide (AlP) intoxication in rat heart. These activities will be detected by histopathological examination and some oxidative stress biomarkers.

**Methods:**

a single sub-lethal dose of Alp (2 mg/kg body weight) was administered orally, and Lam was given orally at a dose (100 mg/kg body weight) one hour after receiving AlP to rats.

**Results:**

aluminium phosphide caused significant cardiac histopathological changes with a significant increase in malondialdehyde (MDA); lipid peroxidation marker; and a significant depletion of antioxidant enzymes (catalase and glutathione reductase). However, treatment with Lam protected efficiently the cardiac tissue of intoxicated rats by increasing antioxidants levels with slight decreasing in MDA production compared to untreated group.

**Conclusions:**

This study suggested that Moringa oleifera extract could possibly restore the altered cardiac histopathology and some antioxidant power in AlP intoxicated rats, and it could even be used as adjuvant therapy against AlP-induced cardiotoxicity.

## Highlights

• Aluminium phosphide (AlP) affects many organs especially heart.
• Antioxidant activities of Moringa was investigated after acute AlP intoxication.
• Moringa preventive effects were determined histopathologically.
• Moringa significantly re-equilibrates antioxidant parameters back near to their normal values.

## Graphical abstract

**Figure:**
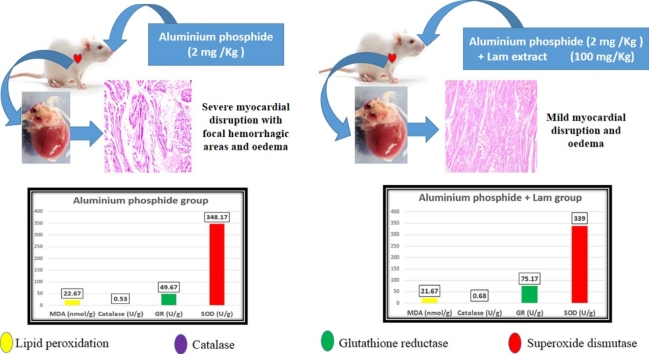

## 1 Introduction

Aluminium phosphide (AlP) is one of agrochemical pesticides that is used to increase agriculture production [1]. Furthermore, it extensively misused as suicidal poison due to low cost availability. In Egypt, AlP is emerging as a common self-poisoning agent [2].

AlP multisystem toxic involvement has been connected with phosphine gas and oxidative stress [3]. Phosphine gas induces oxidative stress through inhibition of enzymatic antioxidants e.g. catalase (CAT), glutathione, glutathione reductase (GR) and superoxide dismutase (SOD) [4]. Inhibition of SOD, CAT and GR will produce superoxide radicals and reduce nitric oxide (NO) bioavailability. The reduced NO level increases neutrophil adherence to coronary vessels with subsequent vasoconstriction. On the other hand, excess superoxide radicals react with NO enhancing lipid oxidation [5,6].These alterations will lead to cellular injury and apoptosis through peroxidation of membrane lipids and disruption of membrane permeability [7,8].

Cardiac tissue is more vulnerable to AlP induced oxidative stress than other human tissues, as it has an elevated oxidative metabolic activity and an increased polyunsaturated fatty acids content [9] [[10], [11], [12]]. To the extent that seventy percent of AlP related deaths were attributable to cardiovascular complication [13]. Impairment of cardiac functions could be detected by several echocardiographic techniques and indices [9].

Moringa oleifera (Lam) is an umbrella shaped tree, and is known as ‘the miracle tree’ due to its health benefit effect [14]. Lam has many natural antioxidant compounds e.g. flavonoids, ascorbic acid, carotenoids, and phenolics. Moringa; as phenolic containing compound, has cardio-protective effect and prevents oxidative myocardial cell damage through enhancing oxidative stress defence enzymes, preventing lipid membrane peroxidation [15,16], and inhibiting the disruption of mitochondrial membrane [17].

Given the evidence that Lam may have a role in managing of AlP acute toxicity, we investigated antioxidant activities of Lam in counteracting the high oxidative stress induced by acute AlP intoxication in rat heart.

## 2 Materials and methods

In this study, the Lam antioxidant activities was detected histopathologically and biochemically through detection of malondialdehyde (MDA) concentration, (SOD), (CAT) and (GR) activities in rat heart. The study was ethically approved by the Institutional Animal Care and Use Committee (IACUC), Cairo University with number (**CU/III/S/41/17).**

### 2.1 Chemicals

Tablet form of aluminium phosphide (3 gm) was purchased from Sandhya Industries Pvt. Ltd., Gujarat, India. While Moringa extract (Lam) was purchased from Egyptian National Research Center (1 gm/mL aqueous preparations).

### 2.2 Animals and experimental design

Twenty-four male Wister rats weighting 100–135 g were used in the study. Animals were housed six cages (four rats/cage), kept under standard laboratory conditions; temperature was 25 ± 2 °C with 40% humidity and allowed free access on commercial diet and tap water provided *ad libitum*.

Rats were divided into three groups with eight animals each. Group I (control) was served as untreated rats and received 0.9% saline solution orally through gastric tube. Group II (AlP intoxicated rats) was given oral single sub-lethal dose of AlP (2 mg/Kg body weight) through gastric tube [18]. Group III (Lam treated group) was given oral single sub-lethal dose of AlP (2 mg/Kg body weight) and oral single dose of Lam (100 mg/Kg body weight) [4] one hour after receiving AlP dose. All groups were observed for 8 h then all rats were sacrificed under pentobarbital anaesthesia by decapitation.

### 2.3 Histopathological examination of heart tissue

Full thickness heart samples from each group were fixed in 10% neutral buffered formalin. The fixed specimens were then trimmed, washed and dehydrated in ascending grades of alcohol, cleared in xylene, embedded in paraffin, sectioned at 4–6U thickness and stained by hematoxylin and eosin dye for photo microscopic examination according to Bancroft et al.[19].

### 2.4 Assessment of oxidative stress biomarkers in heart tissue

Heart specimens were minced and homogenized (10%) in ice-cold 1.155 KCl-0.01 M sodium and potassium phosphate buffer (pH 7.4) in a Potter–Elvehjem glass homogenizer. The homogenate was centrifuged at 10,000 rpm for 20 min at 4 °C, and the resultant supernatant was separated and analyzed to estimate malondialdehyde (MDA) concentration, superoxide dismutase (SOD), glutathione reductase (GR), and catalase (CAT) activities.

Lipid peroxidation, (MDA) level, in heart homogenates was measured spectrophotometrically (Boeco S-20 Spectrophotometer, Hamburg, Germany) using Biodiagnostic kit (Egypt) following Okhawa et al. [20] method.

(CAT) (U/g), (SOD) (U/g) and (GR) (U/g) activities were detected spectrophotometrically (Boeco S-20 Spectrophotometer, Hamburg, Germany) using Biodiagnostic kit (Egypt) following Okhawa et al. [20], Aebi [21], Nishikimi et al. [22] and Goldberg and Spooner [23] respectively.

### 2.5 Statistical analyses

Data were coded and analyzed using the statistical package SPSS version 24. Quantitative variables were presented in mean and standard deviation. Comparisons between groups were done using analysis of variance (ANOVA) with multiple comparisons post hoc test (Chat 2003). P-values less than 0.05 were considered significant.

## 3 Results

### 3.1 Histopathological examination of heart tissue

**Fig. 1:**
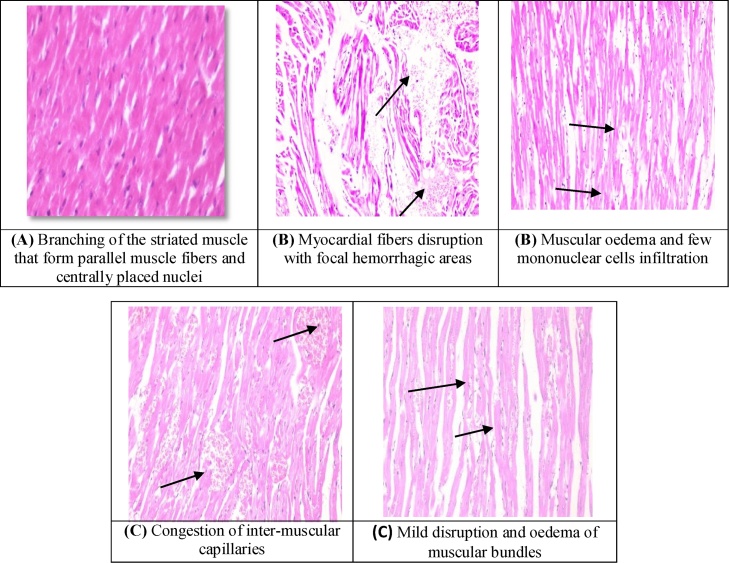
Photomicrograph of heart section in group I (A), group II(B), and group III(C).

Normal cardiac architecture in the form of parallel muscle fibers, centrally placed nuclei, and intercalated discs was presented in Fig. 1(A). However, AlP intoxicated cardiac muscles showed severe fibers disruption with focal hemorrhagic areas between the muscle bundles, muscular oedema and mononuclear cells infiltration. Muscular bundles necrosis and myocytes swelling were also noticed Fig. 1(B). In addition, Lam treated cardiac muscle revealed congestion of inter-muscular capillaries with mild disruption and oedema of muscular bundles Fig. 1(C).

### 3.2 Assessment of oxidative stress biomarkers in heart tissue

The level of malondialdehyde (MDA) was significantly increased in AlP intoxicated and Lam treated groups. The significant highest level of MDA was found in AlP intoxicated group **22.67** **±** **3.50** Table 1.

**Table 1:** oxidative stress and enzymatic antioxidant parameters among different studied groups.

Measured parameters	Name	Group I	Group II	Group III
Oxidative parameter (nmol/g)	Malondialdehyde	**16.83 ± 1.47**	22.67 ± 3.50*	21.67 ± 3.50*
Antioxidant parameters (U/g)	Superoxide dismutase	**319.67±0.82**	348.17 ± 8.01*	339.00 ± 9.42*
	Catalase	***.78±0.01***	.53±0.07*	.68±0.05*^,^#
	Glutathione reductase	***80.93 ± 9.74***	49.67 ± 16.22*	75.17 ± 15.42*^,^#

* statistically significant compared to group I (P < 0.05).

# statistically significant compared to group II (P < 0.05).

While enzymatic antioxidant parameters were significantly decreased in AlP intoxicated and Lam treated groups except GR was significantly increased in Lam treated group. The significant highest levels of CAT and GR were found in Lam treated group 0.68 ± 0.05 and 75.17 ± 15.42 respectively Table 1. Even so, the highest level of SOD was found in AlP intoxicated group **348.17** **±** **8.01.**

## 4 Discussion

The induction of oxidative stress by AlP is well documented [24]. Indeed, AlP significantly increased the main products of lipid peroxidation, MDA, and decreased the activity of CAT and GR, while SOD activity was increased. The explanation for this apparent increase is that the cellular production of antioxidant molecules is increased as a compensatory mechanism against free radicals [9,25].

Many studies [[26], [27], [28]], have reported that the enhanced production of ROS was detected in AlP toxicity. ROS promote membrane lipid peroxidation in cardiac tissue due to presence of polyunsaturated fatty acids and oxygen [9].

In comparison to AlP intoxicated group, the antioxidant activity of Lam was observed. Lam increased CAT and GR activities, while it decreased the MDA level. Moreover, it decreased SOD activity, indicating that the AlP induced oxidative stress was too high [9].

Sheweita et al. [5] has reported that induction of SOD and CAT antioxidant activities is a cellular defence mechanism to withstand oxidative insult. In addition, GR increases the availability of reduced glutathione, which is a cellular antioxidants and NO, which is a vasodilator factor. Lam is capable to reduce lipid peroxidation of cell membrane and prevent free radicals induced damage through its antioxidant activity achieved by its active compounds [8,15,16,29].

Azad et al. [30] has reported that AlP cardio-toxicity can be caused by the oxidation of myocardial cell membranes and internal lipid structure. Moreover, antioxidant therapy has an important role in managing this oxidative insult.

Compared to control animals, AlP intoxicated group were characterized by marked histo-pathological abnormalities in cardiac tissues. These findings were in accordance with the findings of Shah et al. [31] who observed nearly the same histo-pathological changes. The observed AlP effect was evident in Akkaoui et al. [32] and Chugh et al. [33] studies. They reported that AlP intoxication was associated with left ventricular dysfunction, low ejection fraction, severe hypotension and electrocardiographic abnormalities [34,35].

AlP cardiac toxic effect was not found in Anand et al. [13] study. Even so, serum levels of cardiac enzymes were higher in AlP intoxicated rats. The absence of AlP toxic effect could be attributed to their usage of a single lethal dose (20 mg/kg body weight) that caused rapid animal death without eliciting inflammatory response.

While, Lam extract exerted protective effect against AlP–induced cardiac toxicity. It decreased muscle fiber disruption, necrosis, focal hemorrhagic areas between the muscle bundles, mononuclear cells infiltration and myocytes swelling. This is in agreement with Hashemzaei et al. [17] who stated that the polyphenol containing compounds e.g. Lam can stimulate mitochondrial metabolism, enhance the expression of respiratory chain components, and enhance oxygen tissue uptake. Moreover, it also can inhibit the main apoptotic pathway through modulating Akt protein expression.

## 5 Conclusion

In the present study, severe histological alterations were identified in AlP intoxicated group. Furthermore, our data highlighted the oxidative stress as a possible mechanism for AlP induced cardio-toxicity. Treatment with Lam could ameliorate the cardio-toxic effect of AlP due to its contents of antioxidant phytochemicals. Moreover, it has less adverse effects with lower economic burden than commercial drugs [36]. Hence, it can be used as adjuvant therapy in AlP induced cardio-toxicity. However, further echocardiographic studies evaluating cardio-toxic effects of AlP at different concentrations as well as the specific impact of Lam administration on cardiac functions are needed.
